# An Unusual Cause of Fever in a Patient with Total Hip Replacement

**DOI:** 10.7759/cureus.496

**Published:** 2016-02-14

**Authors:** Raju Vaishya, Amit Kumar Agarwal, Vipul Vijay

**Affiliations:** 1 Orthopaedics, Indraprastha Apollo Hospitals

**Keywords:** pyrexia of unknown origin, total hip arthroplasty, drug fever

## Abstract

Pyrexia of unknown origin (PUO) in a patient with acquired immunodeficiency syndrome (AIDS) is a challenging clinical problem despite recent advances in the diagnostic modalities. The diagnosis of the cause of fever is especially difficult in the postoperative period as the focus remains on the operative site. We present an unusual cause of PUO in a patient with advanced HIV disease during an immediate postoperative period following total hip arthroplasty (THA) for osteoarthritis (OA) of the left hip. The fever started on the eighth postoperative day, and after an extensive workup to rule out infection it was found that the patient was allergic to sulfa drugs. The fever subsided after discontinuation of trimethoprim/sulfamethoxazole. Fever in an immunocompromised patient should not be attributed only to infection. A high index of suspicion along with careful history making is required to diagnose drug fever. An early diagnosis of drug fever can reduce hospital stay and the costs of investigations and treatment.

## Introduction

Traditionally, pyrexia of unknown origin (PUO) has been defined as a ‘fever of  38.3 degree Celsius or more lasting more than three weeks and which remains undiagnosed even after one week of inpatient evaluation’ [1]. Lately, the criterion for PUO has been changed to ‘undiagnosed after three outpatient visits or three days of inpatient evaluation’ [2]. The cause of PUO in acquired immuno deficiency syndrome (AIDS) patients is usually infection, inflammatory pathology, malignancy, or sometimes an unknown cause [3]. PUO in AIDS is a challenging clinical problem despite recent advances in the diagnostic modalities [4]. This is especially difficult in the postoperative period as the focus of PUO remains around the operative site. We present an unusual cause of PUO in advance HIV disease during an immediate postoperative period following total hip arthroplasty (THA) for osteoarthritis (OA) of the left hip. Informed patient consent was obtained for this study.

## Case presentation

**Figure 1:**
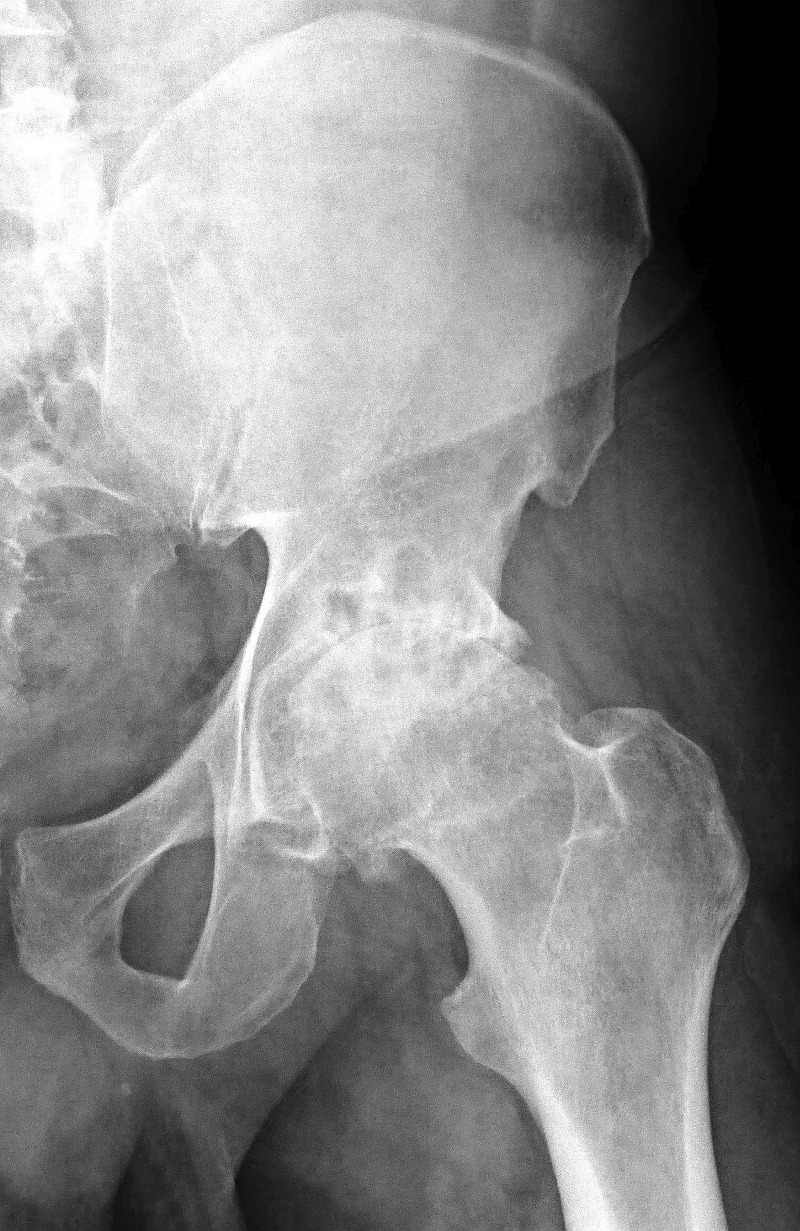
Plain preoperative radiograph (anteroposterior view) of the left hip showing arthritis of hip.

A 58-year-old male from Tanzania presented with a history of progressive left hip pain and stiffness for the last two years. The pain and stiffness was experienced more when he walked, and it had also started affecting his night sleep and daily activities. He was taking diclofenac sodium regularly, but it had stopped giving him any pain relief. He was diagnosed as having severe OA of the left hip, for which a THA was advised. This patient had associated human immunodeficiency virus (HIV) and hepatitis B virus infections, as comorbid conditions. He was not taking any antiretro viral therapy (ART). There was a significant problem of communication due to the language barrier and a lack of desire on his part to disclose his medical problems. The general physical examination was normal, and the local examination of the left hip revealed a fixed flexion deformity of 15 degrees with restriction of hip movements. A plain radiograph of the pelvis confirmed the diagnosis of left hip arthritis (Figure 1).

**Figure 2:**
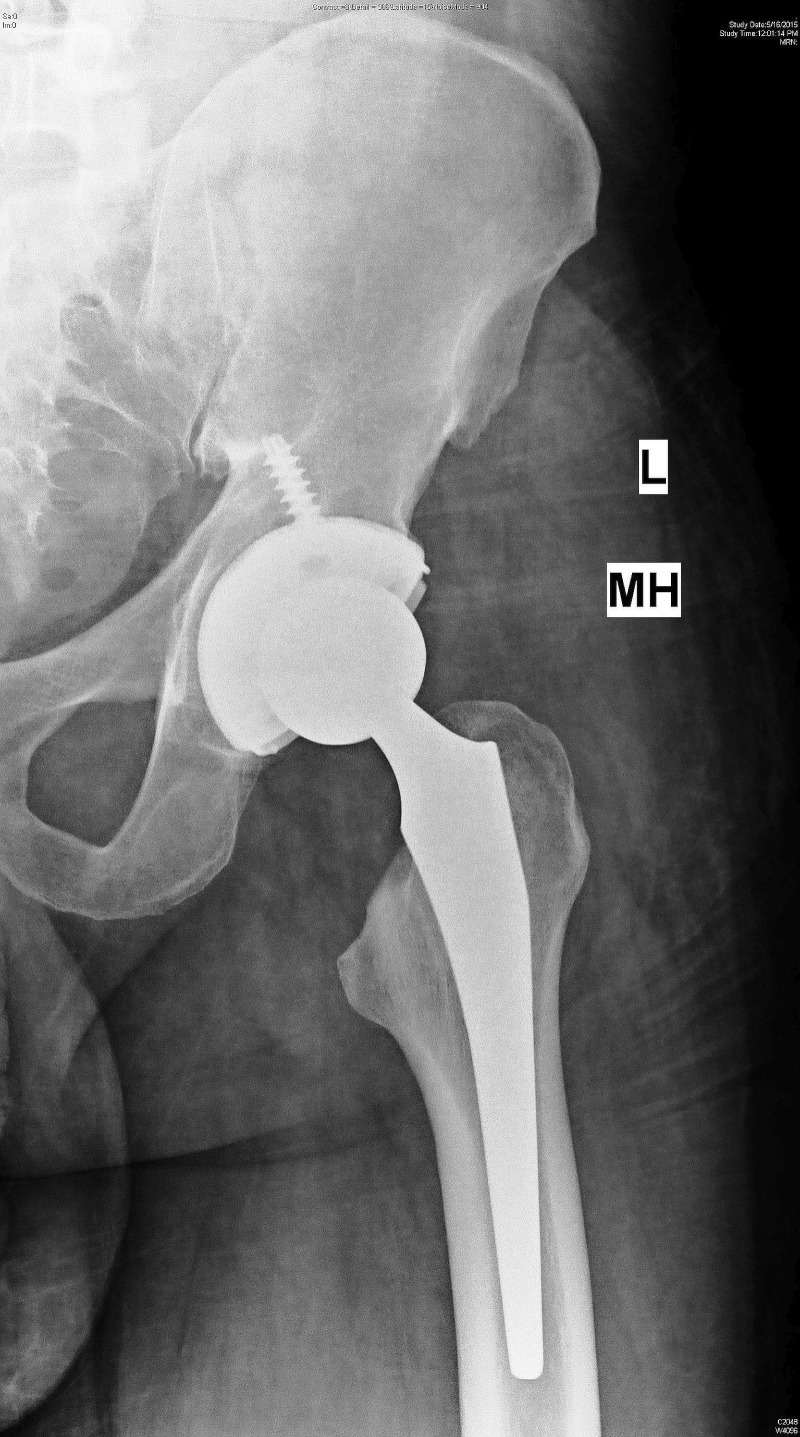
Postoperative radiograph (anteroposterior view) of the left hip showing total hip replacement.

Routine pre-operative laboratory investigations were all within normal limits, except that he had positive rapid tests for HIV and hepatitis B antigen. His HIV-1 RNA titer was 14,500 copies/ml, and HBV DNA titer was 268 IU/ml. The immunodeficiency panel-III showed a significant low absolute count of a low cluster of differentiation 4 (CD4) of 47 (N: 410-1590/ul) and percentage population of CD4 (helper/inducer cells) was only 4% (N: 31-40%). However, the CD3 count was normal. Due to a very low CD4 count, he was counseled against the surgery because of the high risk of infection. However, he insisted on surgery, was willing to take all the risk, and signed a high-risk consent for the left THA. He also promised to take a regular ART after the THA. He underwent left THA under general anesthesia, after all the universal precautions were taken by the surgical team members. The intra- and postoperative period were uneventful. The left THA (Figure 2) was done using uncemented prosthetic components (Accolade Femoral Stem, Tritanium Acetabular Shell with the crossed-linked polyethylene liner and a 36 mm *delta* Ceramic Head) of Stryker™, Michigan.

**Figure 3:**
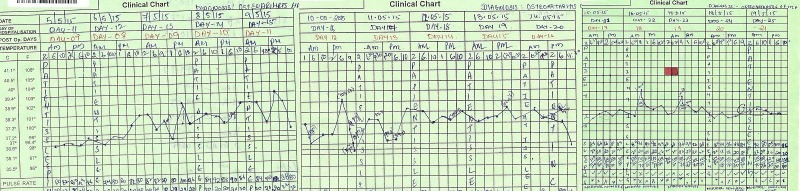
Chart showing the febrile episodes of the patient over a period of time.

He was kept on intravenous (IV) antibiotics (teicoplanin 400 mg once daily and meropenem 1 gm thrice a day) for prophylaxis. His postoperative recovery was satisfactory, and he started mobilizing partial weight bearing on the left leg, with walking aids. His IV antibiotics were discontinued after 72 hrs and were replaced by oral antibiotics (clarithromycin 500 mg twice a day and co-trimoxazole twice a day), for prophylaxis of mycobacterium avium complex (MAC) and other opportunistic organisms. He remained afebrile, and his wound remained dry until eight days after the surgery, when he started having a fever, ranging from 99–103 degree Fahrenheit associated with melena. His upper gastrointestinal endoscopy revealed pan gastritis with no ulcers. The blood and urine cultures did not show any microorganism growth. His blood counts were also not significantly abnormal. The patient was again started on intravenous (IV) antibiotics (teicoplanin 400 mg once daily and meropenem 1 gm thrice daily) for prophylaxis. His pyrexia persisted for eight days (Figure 3).

During this period, repeated blood counts, cultures (blood, urine, and stool) were done which did not give any conclusive evidence for his pyrexia. A peripheral smear for malaria parasite was also negative. A bone marrow aspirate showed moderate cellularity, markedly depressed erythropoiesis but no toxic granules. A Venous Doppler of lower limbs did not show any evidence of deep vein thrombosis. His serum procalcitonin level was raised (6.11 ng/ml). The PCR assay of CMV RT, JC Virus, HBV, and HSV 1 & 2 were negative. An ELISA TORCH complex profile (IgM) was also non-reactive. The surgical site did not show any infection, and the wound had healed by primary intention. He had no pain in the left hip. Since no apparent cause of pyrexia was forthcoming, a computerized positive emission tomography (PET) scan was done, which confirmed no focal peri-implant FDG uptake. There was no significant FDG avid lesion in the body. On the 15th postoperative day, his pyrexia was persisting (Figure 3) and he also developed swelling around the lips, eyelids, and face, and looked toxic. His blood counts showed an increase in the polymorphonuclear count of 90%, deranged liver enzymes (SGOT- 286 U/L, SGPT: 159 U/L) and mild renal dysfunction (creatinine: 1.6, urea: 58) (Table 1).

**Table 1:** Table showing various blood reports done at regular intervals.

Serial No.	Blood Test	Range	Unit	25/04/15 (Pre-op)	07/05/15 (Day-9 Post-op)	10/05/15 (Day-12 Post-op)	14/05/15 (Day-16 Post-op)	18/05/15 (Day-20 Post-op)
1	WBC count	4-11	10^3^/mm^3^	3.4	5.17	3.38	5.95	11.1
2	Neutrophils	45-75	%	52	78	89	90	81
3	Lymphocytes	20-45	%	38	16	07	08	11
4	Monocytes	2-10	%	08	06	02	02	07
5	Eosinophils	1-6	%	02	00	02	00	01
6	Hemoglobin	13-17	g/dl	12.3	8.9	9.3	7.3	9.3
7	ESR	0-15	mm/1^st^ hr	21	100	75	85	60
8	Platelet count	140-440	10^3^/mm^3^	114	381	238	126	214

Due to his deteriorating condition and persistent fever, a review meeting of all involved doctors was organized. As a diagnosis of exclusion, drug fever was considered as the cause of PUO. The patient was taking co-trimoxazole (containing sulfamethoxazole) for the last two weeks, and sulfa drugs are notorious for causing drug fever. Hence, co-trimoxazole was immediately stopped, and he was given IV hydrocortisone (100 mg) and pheniramine (45.5 mg) for three days. His facial erythema and swelling subsided within 12 hours of this drug therapy, and his laboratory markers of blood count, fever, and liver and kidney functions became normal in the next 48 hours. (Table 1)

## Discussion

It is very common for the clinicians to suspect infection as the first cause of fever, and surgery-related infection of the operative site is suspected as the first differential especially in the postoperative period. In patients with AIDS, a fever due to all possible opportunistic infections is often suspected but the lack of a holistic view and an outside-the-box thinking by the treating team, can lead to unnecessary investigations and management of persistent fever. A rarer cause of fever could be related to drugs (drug fever). Although reported to be a not so uncommon cause of fever, it is highly under diagnosed and under suspected. Drug fever (DF) is a febrile response coinciding with the drug administration in the absence of any other underlying conditions [5]. A characteristic feature of DF is that it usually disappears once the offending drug is discontinued [6]. It is a diagnosis of exclusion, and it is often suspected in patients with otherwise unexplained fevers and for whom infection has been ruled out [7]. An awareness of this entity and a high index of suspicion is required by the treating physicians to diagnose DF. Clinicians must be aware of the common offending drugs that may cause DF to avoid unnecessary and costly diagnostic workup, incorrect treatment with multiple antibiotic therapy, and longer hospital stays [8]. Suspecting infection in a condition where an infection is not the cause of fever leads to overutilization of antibiotics increasing the risk of unwarranted side effects and the development of antimicrobial resistance [9]. It is important to diagnose DF early during its course as it may be accompanied by more severe adverse drug reactions if not diagnosed in time [10]. Sometimes DF and infection may coexist and thereby give a false impression that a successful course of antibiotic therapy is failing.

A literature search has found that there are variations amongst various drug classes between the initiation of the offending drug and the onset of fever [11]. During the drug therapy, DF can occur at any point in time. In our patient sulfa drug (co-trimoxazole-trimethoprim/sulfamethoxazole) was the responsible drug and the fever started after three days of starting this drug. Co-trimoxazole was begun as a prophylactic drug in an HIV-infected patient for mycobacterium avium complex (MAC) and other opportunistic organisms.

Though various patterns of DF fever are possible (e.g., continuous, remittent, intermittent, and hectic), the hectic pattern is the most common presentation [12]. However, the use of antipyretics can change the natural pattern of fever. Moreover, the degree of fever may vary from low to high grade. In our patient, the fever ranges from 99–103 degree Fahrenheit, and it was remittent in type as it was never coming to baseline.

Although blood investigations can be helpful in the diagnosis of DF, they are highly variable. A raised total white cell count (WCC) with or without a left shift may be associated with DF. An elevation of erythrocyte sedimentation rate (ESR) is also sometimes found in drug fever [13]. No single laboratory test is consistent with the diagnosis of DF. In our case, the blood tests were noncontributory and only ESR was persistently elevated during the febrile episodes.

There are various mechanisms of DF, but hypersensitivity reaction is considered as the most common mechanism of DF. Other mechanisms include the effects of thermoregulation, idiosyncratic response, drug administration-related reactions, and the pharmacologic action of a drug. It is often difficult to pinpoint any exact mechanism of DF in clinical practice.

The variation in the time of onset, duration, and mechanism of fever, apart from a wide variety of drugs causing fever makes it often difficult to reach a reasonable and early conclusion. It is a challenge for the clinicians to decide whether to continue the suspected offending drug, keeping in view the risk-benefits ratio. There is no denial of the fact that a careful history making cannot be substituted, and it is imperative that all treating staff members should highlight any drug allergies clearly if at all present in any of the documents. A warning (red) band should be put on the patient’s wrist at the time of admission to make it apparent to all the treating team at all times. The patients should be encouraged to reveal all the relevant history; for at times they tend to hide the history, especially if they have acquired HIV or a similar infection with social stigma attached to it. We also suggest that the pharmacovigilance team of the hospital be equally cautious and point out to the treating physician for possible discontinuation, the drugs with the highest index of suspicion of causing the fever.

## Conclusions

Pyrexia of unknown origin (PUO) during an immediate postoperative period in a patient with AIDS is a challenging clinical problem despite recent advances in the diagnostic modalities. Fever in an immunocompromised patient should not be attributed only to infection. A high index of suspicion along with careful history making is required to diagnose drug fever, after excluding infection. An early diagnosis of drug fever can reduce morbidity, and costs of investigations and treatment.
